# Patterns of unhealthy behaviours during adolescence and subsequent anxiety and depression in adulthood: a prospective register linkage study of the HUNT survey and health registries

**DOI:** 10.1186/s12966-023-01408-2

**Published:** 2023-01-23

**Authors:** Annette Løvheim Kleppang, Mario Vianna Vettore, Ingeborg Hartz, Siri Håvås Haugland, Tonje Holte Stea

**Affiliations:** 1 Department of Health and Nursing Science, University of Agder, Kristiansand, Norway; 2 Innlandet Hospital Trust, Hedmark, Norway; 3 Department of Health and Nursing Science, Inland Norway University of Applied Sciences, Elverum, Norway; 4 Department of Psychosocial Health, University of Agder, Kristiansand, Norway

**Keywords:** Health-related behaviours, Physical activity, Diet, Sleep, Depression, Anxiety, Latent class analysis, Prospective register linkage study

## Abstract

**Background:**

There is increasing need for prospective investigations in the preventing role of health-related behaviours on mental health problems. The aim of this study is to identify patterns of health-related behaviours in adolescence, and the association between the behavioural patterns and the subsequent diagnoses and/or drug treatment for anxiety and/or depression in adulthood.

**Methods:**

This prospective study consisted of 13–19-year-old participants in the Trøndelag Health Study (Young-HUNT3) in 2006–2008 (*n* = 2061, 1205 females and 856 males) in Norway, who also participated in HUNT4 (2017–2019). Survey data on health-related behaviours in adolescence, including low level of physical activity, low consumption of wholegrain bread, fish, fruit, vegetables and high consumption of sugar-sweetened beverages and insomnia were linked on an individual level to prospective information on drug use and diagnosis in national health registries. The different patterns of health-related behaviours were identified through latent class analysis. Subsequent anxiety or depression was defined as at least one recording in either of three registries covering recorded diagnosis in primary and specialist healthcare, or dispensed prescription drugs during 2008–2019. Additionally, self-reported psychological distress measured in young adulthood was applied as a supplemental outcome measure.

**Results:**

Four patterns of health-related behaviours were identified: high risk behaviours (class 1), moderate to high risk behaviours (class 2), low to moderate risk behaviours (class 3) and low risk behaviours (class 4). Adolescents in class 3 showed higher odds of subsequent diagnoses for anxiety and/or depression in primary and specialist healthcare compared to class 4 participants. In addition, both class 1 and class 4 participants had higher odds for self-reported psychological distress than those class 4 (OR = 1.56 and OR = 1.86, respectively).

**Conclusions:**

Our findings suggest that health-related behaviours are clustered among Norwegian adolescents. The patterns of unhealthy behaviours during adolescence only partly increased the risk of anxiety and depression in adulthood. Promoting healthy behaviours during adolescence may potentially reduce the burden of mental illness in adulthood, but further research is needed to clarify the nature of the relationships.

## Background

The prevalence of mental distress and illness has been increasing, and anxiety and depression are among the most common psychiatric disorders worldwide [1–4]. A systematic review and meta-analysis, which included information from 32 different countries conducted in Africa, America, South-East Asia, Europa, Eastern Mediterranean and Western Pacific WHO regions, reported that the overall pooled prevalence of psychological distress, anxiety and depression was 50.0, 26.9 and 28.0%, respectively [5]. According to estimates for prevalence rates of common and severe mental disorders in Europe, Norway had higher rates than central and eastern European countries, but lower than Ireland and Portugal [6]. The early onset of mental disorders has been associated with a considerably increased risk of mental health problems throughout one’s lifespan [7, 8]. Moreover, a German study has reported that over a 12-month period, almost half of those who have a mental disorder experienced a comorbidity of several other mental disorders [9]. From a public health perspective, it is therefore urgent to identify modifiable factors associated with increased risk of poor mental health to develop tailored measures that can prevent and treat mental disorders.

Despite a possible strong link between unhealthy lifestyle behaviours and poor mental health, global studies as well as Norwegian studies, have reported that few adolescents adhere to guidelines for physical activity [10, 11], food and beverage consumption [12, 13] and sleep [14, 15], provided by international and national health authorities.

A recent overview on this topic suggested that physical activity, healthy diet and restful sleep are independently associated with a lower risk for certain mental disorders [16]. Likewise, a recent systematic review concluded that health behaviours, including regular physical activity, not smoking, healthy diet and restful sleep, may play an important role in the prevention and treatment of mental illness [17].

A systematic review and meta-analyses of prospective studies have also reported an association between low and medium cardiorespiratory levels and increased risk of common mental health disorders compared to those with high cardiorespiratory levels [18]. In addition, a 13-year cohort study reported that mentally-active sedentary behaviours (e.g., office-work) appears to reduce the risk of depression onset and low and medium cardiorespiratory levels are associated with a greater risk of common mental health disorders than high cardiorespiratory [19].

Low quality diet has also been associated with mental health problems early in one’s lifespan [20]. Furthermore, an inverse association between a healthy diet and risk of depression was reported, but the results are of moderate quality [21]. Further evidence suggests that poor diet is a relevant predictor in the onset of depressive illness [22], whereas healthy eating patterns may reduce anxiety symptoms [23]. The consumption of specific food items and beverages, such as high consumption of fruit, whole grain bread and fish and low consumption of sugar-sweetened beverages, was also associated with lower levels of depressive symptoms in adolescents [24] and lower risk of mental distress among adults [25]. Some studies suggest an association of higher consumption levels of proinflammatory diets and Western diets with the increased incidence of depression, while higher intake of fruits and vegetables was associated with lower incidence of depression [26]. Moreover, several studies have concluded that the consumption of soft drinks was a meaningful risk factor for developing depression among adults [27–29].

Poor sleep was also documented as a risk factor of later mental health problems, and insomnia, including short sleep duration and sleep quality, was a significant predictor for the onset of anxiety and depression [30]. Insomnia in adolescence is common [31], and frequently exists along with psychiatric disorders. A recent registry linkage study among children and adolescents concluded that 48% of those with depression reported insomnia [32]. Targeting sleep difficulties at an early stage of life has been suggested as a preventive strategy for the onset of clinical mental disorders [33]. Future research should examine the relationship between sleep habits in adolescence and anxiety and depression later in life [33].

Although the association between different health-related behaviours and later mental health is well documented, information is limited on the association between patterns of unhealthy behaviours, such as low level of physical activity, poor diet and insomnia, in adolescence and subsequent anxiety and depression during adulthood. Latent class analysis (LCA) has become a common approach. Recent studies have suggested that health-related behaviours should be analysed considering a clustering perspective of different behaviours [34, 35]. Previous studies have indicated that different health behaviors are often interrelated, changes in one health behaviour may lead to changes in other behaviours, and also occur concomitantly [36–40]. Therefore, methodological approaches based on co-occurrence of behavioural characteristics is important. Latent class analysis (LCA) is a robust approach which is used to understand classes/construct associated with patterns of factors reported by individuals [41]. This method has become increasingly popular among psychological researchers to enhance the understanding of individual and within-group differences that are not directly observed [42]. LCA provides useful information to analyze the clustered characteristic of unhealthy behaviours and has previously been used to provide information about the relationship between lifestyle behaviors and health status [43, 44]. In real life, different combinations and clusters of behavioural factors may exist, and robust evidence on how different combinations of these factors in adolescence associate with later depression and/or anxiety are essential to improve the ability to identify those at risk of developing a mental disorder and inform health promotion initiatives. In Norway, information on all contacts with and diagnosis set in primary and specialist health care, and all dispensed prescription drugs for anxiety and depression, and other diagnoses, are available in nationwide health registers. Thus, we have the unique opportunity to link information on health-related behaviours at individual level from population-based surveys to nationwide health registries for prospective information on diagnosis and drug treatment for anxiety and depression [45]. The present study aimed to investigate the influence of different patterns of adolescent risky health-related behaviours on subsequent diagnoses and/or drug treatment for anxiety and/or depression in adulthood.

## Methods

**Fig. 1:**
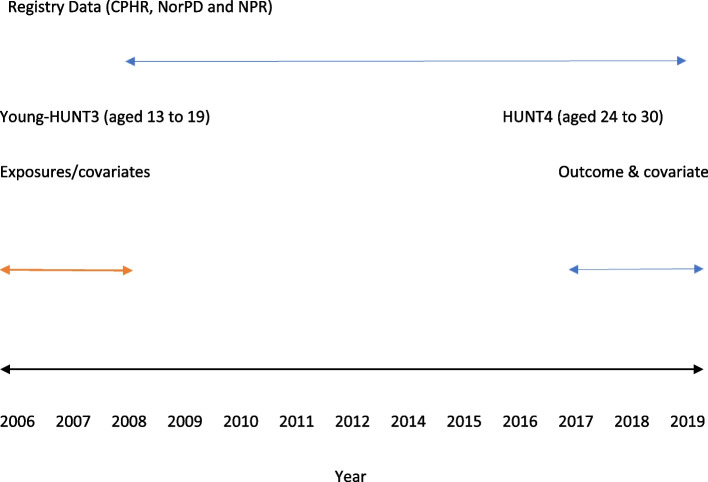
Longitudinal design of the study and use of different data sources

The present longitudinal prospective study was conducted using linked survey and health registry data in Norway. Our analytical sample consisted of 2293 participants from Norway who participated in the adolescent version of the survey of the Trøndelag Health Study (Young-HUNT3) in 2006–2008 when they were aged 13–19 years and then later in the adult’s part of the survey in 2017–2019 (HUNT4) when they were aged 23–31 [46]. Finally, after excluding those with missing data on any of the lifestyle items (*n* = 232), the current study comprised 2061 respondents (1205 females and 856 males). This sample was then linked with data from three national health registries from 2008 to 2019. Data from the different sources were linked at the individual level using the personal identification number assigned to all Norwegian Citizens. The overview of the study design and data sources are described in Fig. 1.

The HUNT study is a collaboration between the HUNT Research Centre (Faculty of Medicine and Health Sciences, Norwegian University of Science and Technology NTNU), Trøndelag County Council, the Central Norway Regional Health Authority and the Norwegian Institute of Public Health.

Participation in the studies was voluntary, and the respondents were informed that they could withdraw from the studies any time. The present study was approved by the Regional Committees for Medical and Health Research Ethics (No. 101672) and the Norwegian Data Protection Authority (No. 308702).

### Measures and data sources

*Young-HUNT3 (2006–2008) and HUNT4 (2017–2019)*. Data on exposures of interest and other substantive covariates was gathered from the Young-HUNT3 and HUNT4 surveys. The HUNT surveys are a carried out in Trøndelag county, Norway, covering a range of health-related topics. All adolescents in Trøndelag county aged 13 to 19 years were invited to participate in the Young-HUNT3 survey, of whom 8200 adolescents participated (78.4% of all invited). In 2019, 11 years later, the same population was invited to participate in the adult part (HUNT4), of whom 2293 young adults participated, with 1320 (57.6%) females and 973 (42.4%) males.

#### Primary exposure variables (young-HUNT3): health-related behaviours in adolescence

Patterns of health-related behaviours in adolescence were assessed according to the reported frequency of physical activity, consumption of wholegrain bread, fish, fruit, vegetables and sugar-sweetened beverages and insomnia, which were evaluated as criteria for healthy behaviour. The cut-off values used to identify healthy behaviours was based on adherence to national recommendations for physical activity and nutrition [47].

#### Physical activity

Adolescents’ physical activity was assessed according to the question: ‘Outside school hours, how many hours do you usually exercise in your free time so much that you get out of breath or sweat?’ The response options were as follows: ‘never’, ‘approximately 30 min a week ‘approximately 1–1 ½ hours a week’, ‘approximately 2–3 hours a week’, ‘approximately 4–6 hours a week’ or ‘approximately 7 hours and more per week’. A high level of physical activity was defined as ‘four hours or more per week’ (reference category), and ‘less than 4 hours’ was defined as a low level of physical activity, as used in a previous study [48].

#### Consumption of wholegrain bread, fish, fruit, vegetables and sugar-sweetened beverages

Consumption of whole grain bread, fish, fruit and vegetables was measured by the following question: ‘How often do you eat the items listed below?’ The response options were ‘several times a day’, ‘once a day’, ‘every week but not every day’, ‘less than once a week’ and ‘never’. Consumption of sugar-sweetened beverages was measured by the following: ‘How often do you drink the items listed below?’ The response alternatives were ‘seldom/never’, ‘1–6 glasses a week’, ‘1 glass a day’, ‘2–3 glasses a day’ and ‘4 or more glasses a day’. Consumption of foods and beverages was dichotomised into ‘daily consumption and more’ (reference category for foods) and ‘less than daily consumption’ (reference category for sugar-sweetened beverages), as used in a previous study [49].

#### Insomnia

Insomnia was assessed based on the following two questions: ‘Have you had problems falling asleep during the last month?’ and ‘During the last month, did you ever wake up too early, not being able to fall asleep again?’ The following response options were given: ‘almost every night’, ‘often’, ‘occasionally’ and ‘never’. The participants were classified with insomnia if they answered ‘often’ or ‘almost every night’ on at least one of the questions and with no insomnia if they answered ‘less than often’ (reference category) in both questions, as suggested by previous studies [50, 51].

#### Outcomes

##### Main outcome: later diagnosis or drug treatment for anxiety and/or depression (registry data)

Information on the diagnosis and drug treatment for depression and/or anxiety from 2008 to 2019 was obtained through the participants’ records in three nationwide health registries in Norway: 1) the Norwegian Prescription Database (NorPD), which encompasses information of all dispensed prescription drugs to patients in ambulatory care in Norway [52]; 2) the Control and Payment of Health Reimbursements Registry (CPHR) for practitioners in primary health care, which provides information on the International Classification of Primary Care diagnosis code recorded at each visit in primary health care [53]; and 3) the Norwegian Patient Registry (NPR), which has information from admissions to hospitals and other specialist health care and which includes International Statistical Classification of Diseases and Related Health Problems, Tenth Revision diagnosis codes [54]. The details of drugs and diagnoses related to anxiety and depression included in the current study are described in Table 1.

The main outcome measure was defined as follows; at least one recording in either of the three health registers with a drug or a diagnosis described in Table 1 during the defined study period (2008–2019).

##### Secondary outcome: later psychological distress in young adulthood (HUNT4)

Psychological distress was assessed using the CONOR Mental Health Index (CONOR-MHI) in HUNT4. The CONOR-MHI is a modified version of the General Health Questionnaire [55] and Hopkin’s Symptoms Checklist [56], containing seven items related to various aspects of psychological distress. Individuals were asked if during the past 14 days, they have felt ‘nervous and unsettled’, ‘troubled by anxiety’, ‘secure and calm’, ‘irritable’, ‘happy and optimistic’, ‘sad/depressed’ or ‘lonely’. Each item was answered on a four-point Likert scale, ranging from ‘no’ (1) to ‘very’ (4). CONOR-MHI scores were computed by dividing the total score by seven (number of items). Missing values were replaced by the sample mean value for each item. However, records with two or more missing items were excluded (Fig. 1). In the present study, a cut-off point at ≥2.15 was used to determine psychological distress, which is considered a valid threshold for predicting psychological distress [57].

#### Confounders (YoungHUNT3)

##### Gender and age of the adolescents

The age of the adolescents (YoungHUNT3) varied between 12.0 and 20.9 years and 58.5% were females (Table 2).

##### Psychological distress in adolescence

In the YoungHUNT3, psychological distress was assessed by the Hopkins Symptom Checklist-5 (HSCL-5), which is a five-item shortened version of the HSCL-25. The HSCL-25 is a screening tool designed to measure symptoms of depression and anxiety [58]. The HSCL-5 has been shown to be a reliable and valid short form of the HSCL-25 as a screening instrument for symptoms of depression and anxiety [59, 60]. The adolescents were asked if during the past 14 days, they had been affected by the following: ‘been constantly afraid and anxious’; ‘felt tense or uneasy’; ‘felt hopeless about the future’; ‘felt dejected or sad’; or ‘worried too much about various things’. The five items have four response options, ranging from ‘not bothered’ (1) to ‘very much bothered’ (4). The total HSCL-5 score comprised the sum of the items, and the mean score was used as a measure of psychological distress. In the present study, a cut-off point at > 2.0 was used to determine psychological distress, which is considered a valid cut-off value for predicting psychological distress in adolescents [60]. We excluded participants who had missing values on at least one item of the HSCL-5 scale.

##### Highest education as young adults (HUNT4)

Education was assessed by the following question (HUNT4): ‘What is your highest level of education?’ The following response options were used: ‘primary school’, ‘high school’, ‘college ≤4 years’ and ‘college >4 years’. Educational level was dichotomised into ‘no higher education’, which included primary school and high school, and ‘higher education’, which included college ≤4 years and college >4 years (reference category).

The exposure variables were assessed in 2006–2008 (Young-HUNT3,) (Fig. 1). The available years of the three national health registry data and HUNT4 are shown on the right-hand side. Even though some registries were available prior to 2008, the start of the study period was defined in 2008 because that was the first year when all three registries were available and the last year of exposure data collection. This approach ensured equal follow-up time for all participants and equal contribution of information from all three registries.

Table 1 shows an overview of registry entries extracted to identify anxiety and depression problems.

**Table 1:** Overview of Registry Entries Extracted to Identify Anxiety and Depression Problems

***Control and Payment of Health Reimbursements Registry***	
*ICPC* codes	
P01	Feeling anxious/nervous/tense
P03	Feeling depressed
P73	Affective psychosis
P74	Anxiety disorder/anxiety state
P76	Depressive disorder
P79	Phobia/compulsive disorder
**Norwegian Prescription Database**	
ATC codes	
N05AH03^a^	Olanzapine
N05AH04^a^	Quietapine
N05AN01^a^	Lithium
N05AX12^a^	Aripiprazole
ATC codes starting with	Anxiolytics
N05B	
ATC codes starting with	
N06A	Antidepressives
**Norwegian Patient Registry**	
ICD-10 codes	
F30-F39	Mood (affective) disorders
F40-F48	Anxiety, dissociative, stress-related, somatoform, and other nonpsychotic mental disorders

*Abbreviations: ATC *Anatomical Therapeutic Chemical, *ICD-10 *International Statistical Classification of Diseases and Related Health Problems, Tenth Revision, *ICPC *International Classification of Primary Care

^a^Only included if the reimbursement code suggested that the medication was given for mood-related or anxiety-related problems

### Statistical analysis

The participants’ characteristics were described through frequencies, proportions and means with their respective 95% confidence intervals.

The different patterns of health-related behaviours were determined through latent class analysis (LCA) using the maximum likelihood method, which considers maximal interclass differences and minimal intraclass differences [61]. LCA is suitable for dichotomous variable and also enables the identification of distinct configuration of heterogeneity within a given population sample [41], it considers profile membership as an unobserved categorical variable indicating which profile an individual belongs to with a certain degree of probability. In the Young-HUNT3 study, classes of health-related behaviours were estimated according to low and high physical activity, low and high consumption of wholegrain bread, fish, fruit, vegetables, and sugar-sweetened beverages, and presence or absence of insomnia.

A sequence of six LCA models was fitted to identify an optimal baseline model. The best-fitting model was obtained according to Akaike information criterion (AIC) and Bayesian information criterion (BIC) [41], and smaller numbers represent more parsimonious and well-fitting models [62, 63]. The posterior class probabilities and corresponding class memberships were used to assign each participant to a class based on the maximum posterior probabilities of the selected model. The different patterns of health-related behaviours were presented according to the conditional probabilities of the corresponding behaviours. The likelihood ratio test was used to evaluate the goodness of fit of the final model.

Multivariable logistic regression analysis was used to examine the associations between unhealthy-related behaviours (defined by risk classes through LCA analysis) and later diagnosis or drug treatment for anxiety and/or depression (at least one recoding of the three registers). The regression analysis was also conducted according to different registers and self-reported psychological distress (CPHR, NorPD, NPR and CONOR-MHI). All models were adjusted for gender, age and psychological distress in adolescence and highest education in young adulthood. Modification effect analysis was conducted according to the four outcomes to examine the influence of gender and psychological distress on the relationship between health-related behaviours and subsequent depression or/and anxiety and self-reported psychological distress. Possible interaction effects were examined using LR-tests (Likelihood ratio test), contrasting models with and without interaction terms. Two significant interaction effects were detected (gender by CPHR and HSCL-5 by CPHR). Therefore, only these models were examined further (Table 4). Collinearity was also examined using Variance inflation factor (VIF) statistics with a cut-off value of > 10, indicating that multicollinearity was not present. The results were presented as odds ratios (OR) with 95%CI. The LCA were performed using Stata (StataCorp, version 17), all other statistical analyses were performed using SPSS 25.0. The significance level established for all analyses was 5% (*p* ≤ 0.05).

## Results

### Sample description

The sample comprised a total of 2061 participants: 58.5% females and 41.5% males. On average, adolescents were 16 years old when they participated in Young-HUNT3 (Table 2). In adolescence, 13.2% of the respondents reported psychological distress, while in young adulthood, 12.4% reported psychological distress.

Between 2008 and 2019, 33.4% of the participants had at least one recording of a diagnosis or prescription drug in either of the three registries. Most patients had at least one recording in the Control and Payment of Health Reimbursements Registry (26.4%) and the Norwegian Prescription Database (20.1%), and fewer patients had a recording diagnosis in the Norwegian Patient Registry (16.1%).

### Description of LCAs

Table 2 shows the sample characteristics and study variables according to the selected class analysis.

**Table 2:** Sample Characteristics and study variables according to the selected Latent Class Analysis (LCA) solution

Charateristics	n (%; 95% CI)	Mean (SD), range				
			Class 1	Class 2	Class 3	Class 4
Number of participants	2061					
Females^a^	1205 (58.5)		188 (15.6; 13.6-17.7)	398 (33.0; 30.4-35.7)	301 (25.0; 22.5-27.4)	318 (26.4; 23.9-28.9)
Males^a^	856 (41.5)		125 (14.6; 12.2-17.0)	344 (40.2; 36.9-43.5)	197 (23.0; 20.2-25.8)	190 (22.2; 19.4-25.0)
Age at Young-HUNT3 participation, Y^a^		16.0 (1.77), 12.0 – 20.9				
*Adolescent self-reported psychological distress (HSCL-5)*^*a*^						
Higher level of psychological distress	273 (13.2)		59 (21.6; 16.7-26.5)	110 (40.3; 34.5-46.1)	70 (25.6; 20.5-30.8)	34 (12.5; 8.5-16.4)
*Educatinal level in adulthood*^*b*^						
Lower secundary school	28 (1.4)		10 (35.7; 18.0-53.5)	7 (25.0; 9.0-41.0)	9 (32.1; 14.8-49.5)	2 (7.1; 2.4-16.7)
Upper secundary school (1-2 year)	152 (7.4)		45 (29.6; 22.4-36.9)	45 (29.6; 22.4-36.9)	43 (28.3; 21.1-35.5)	19 (12.5; 7.2-17.8)
Upper secundary school (3 year)	325 (15.8)		50 (15.4; 11.5-19.3)	121 (37.2; 32.0-42.5)	81 (24.9; 20.2-29.6)	73 (22.5; 17.9-27.0)
Vocaional education	494 (24.0)		100 (20.2; 16.7-23.8)	200 (40.5; 36.2-44.8)	122 (24.7; 20.9-28.5)	72 (14.6; 11.5-17.7)
Higher education less than 4 year	654 (31.8)		74 (11.3; 8.9-13.7)	243 (37.2; 33.5-40.9)	149 (22.8; 19.6-26.0)	188 (28.7; 25.3-32.1)
Higher education, 4 year and more	402 (19.6)		33 (8.2; 5.5-10.9)	124 (30.8; 26.3-35.4)	92 (22.9; 18.8-27.0)	153 (38.1; 33.3-42.8)
*Adulthood self-reported psychological distress (CONOR MHI)*^*b*^						
Higher level of self-reported psychological distress^b^	255 (12.4)		53 (20.8; 15.8-25.8)	91 (35.7; 29.8-41.6)	69 (27.1; 21.6-32.5)	42 (16.5; 11.9-21.0)
*Registry based outcomes*^*c*^						
At least one recording of the three registries (2008-2019)	689 (33.4)		118 (17.1; 14.3-19.9 )	243 (35.3; 31.7-38.8)	183 (26.6; 23.3-29.9)	145 (21.0; 18.0-24.1)
CPHR (2008-2019)	554 (26.4)		100 (18.1; 14.9-21.3)	190 (34.3; 30.3-38.3)	160 (28.9; 25.1-32.7)	104 (18.8; 15.5-22.0)
NorPD (2008-2019)	415 (20.1)		71 (17.1; 13.5-20.7)	150 (36.1; 31.5-40.8)	115 (27.7; 23.4-32.0)	79 (19.0; 15.3-22.8)
NPR (2008-2019)	332 (16.1)		63 (19.0; 14.8-23.2)	118 (35.5; 30.4-40.7)	95 (28.6; 23.8-33.5)	56 (16.9; 12.8-20.9)

Abbreviations: *CPHR* Control and Payment of Health Reimbursements Registry, *HUNT* Nord-Trøndelag Health Study, *NA* not applicable, *NorPD* Norwegian Prescription Database, *NPR* Norwegian Patient Registry, *HSCL-5* Hopkins Symptom Checklist-5, *CONOR MHI* Conor Mental Health Index (7 items). ^a^Obtained from Young-HUNT3. ^b^Obtained from HUNT4. ^c^For registry-based outcomes

**Fig. 2:**
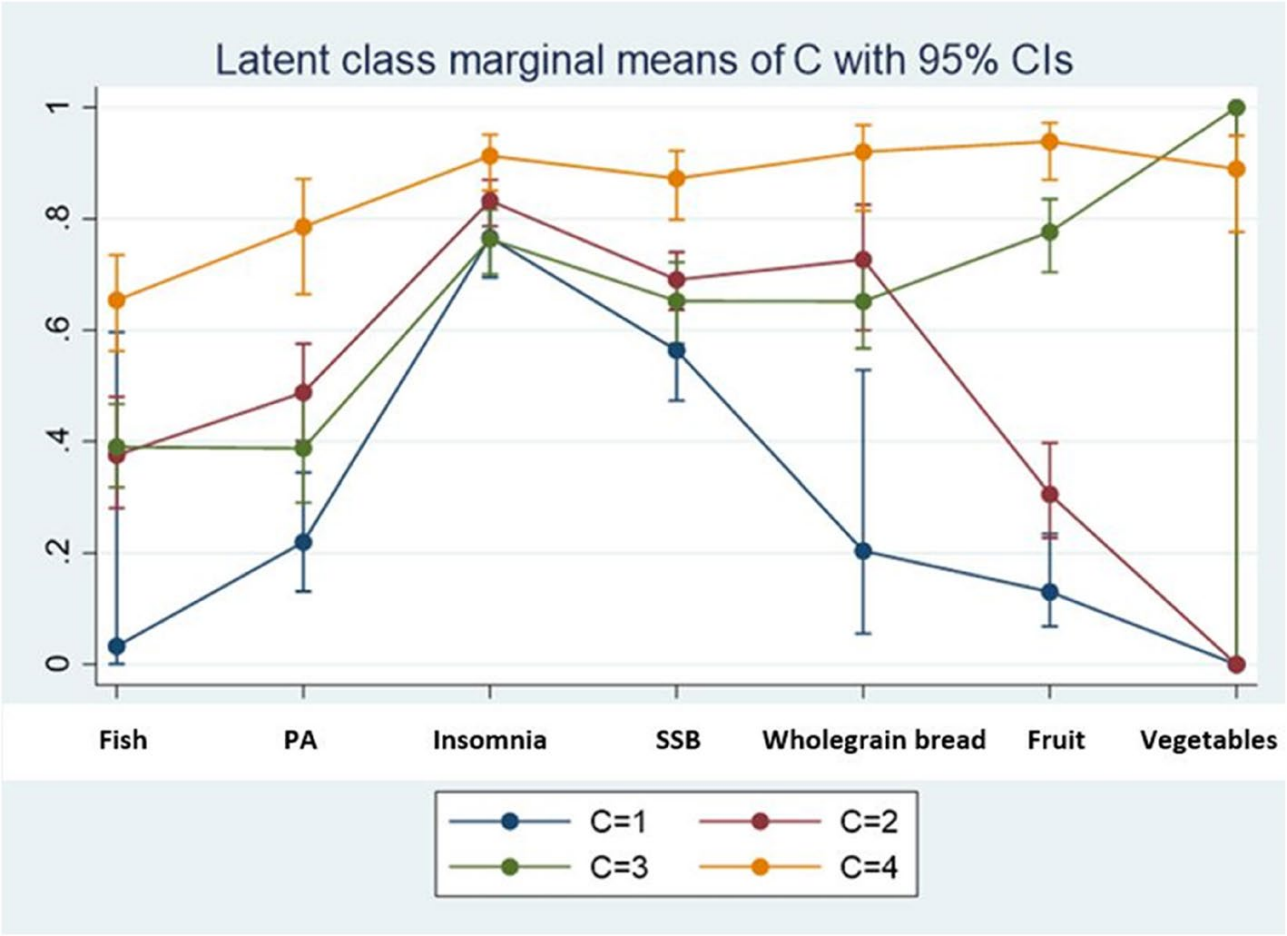
Item-response probabilities of the four latent class models (LCA analysis). The Y-axis indicates the probability of reporting the selected health-related behaviours according to each class. The number for the latent class solution is based on the Bayesian information criterion (BIC), Akaike information criterion (AIC) and likelihood ratio test

Figure 2 shows the probabilities of health-related behaviours for each of our classes (the class characteristics).

Figure 2 presents the item-response probabilities of the four LCA groups. Six latent classes were statistically fitted from two to seven latent classes using LCA analysis. The four-class model was considered the best solution of latent health-related behaviours patterns based on the fit indices, marginal probabilities and conceptual relevance. Class 1 (blue line) labelled the high-risk group of unhealthy behaviour patterns (15.2%), represents adolescents with the highest probabilities of low consumption of fish, low levels of physical activity, insomnia, high consumption of sugar-sweetened beverages, low consumption of wholegrain bread and fruits, and no probability of low consumption of vegetables. Participants in class 2 (red line), with moderate to high-risk of unhealthy behaviour patterns (24.2%), showed a moderate probability of low consumption of fish, low levels of physical activity, higher probability of insomnia, moderate to high probability of high consumption of sugar-sweetened beverages and low consumption of wholegrain bread, moderate to low probability of low consumption of fruits no probability of low consumption of vegetables. Class 3 (green line) was labelled as low to moderate risk of unhealthy behaviour patterns (36.0%) and included those with moderate probability of low consumption of fish, low levels of physical activity, insomnia, moderate to high probability of high consumption of sugar-sweetened beverages, low consumption of wholegrain bread and fruits, and higher probability of low consumption of vegetables. Subjects in class 4 (orange line), included those with healthiest behaviours (24.6%), including a lower probability of low consumption of fish, low levels of physical activity and insomnia, high consumption of sugar-sweetened beverages, low consumption of wholegrain bread and fruits and moderate to higher probability of low consumption of vegetables. Class 4 was considered as the reference group in order to examine the possible association between unhealthy behaviours and outcomes, including at least one recoding of the three registers, CPHR, NorPD, NPR and CONOR-MHI.

Table 3 shows the LCA of health-related behaviours in adolescence that may increase the risk of psychological distress and subsequent anxiety and depression in adulthood.

**Table 3:** Lifestyle habits in adolescence and subsequent contacts with health care system for depression and/or anxiety

Vaiables	At lest one recoding in either of the three registries ^a^	At lest one recoding in either of the three registries ^a^	Supplemental Models							
			Model 1^a^	Model 2	Model 3^a^	Model 4	Model 5^a^	Model 6	Model 7^a^	Model 8
			CPHR	CPHR	NPR	NPR	NorPD	NorPD	CONOR MHI	CONOR MHI
	OR (95% CI)	AOR (95% CI)	OR (95% CI)	AOR (95% CI)	OR (95% CI)	AOR (95% CI)	OR (95% CI)	AOR (95% CI)	OR (95% CI)	AOR (95% CI)
***Latent class analysis of probabiity of lifestyle risk behaviors***										
Class 4	1 (ref)	1 (ref)	1 (ref)	1 (ref)	1 (ref)	1 (ref)	1 (ref)	1 (ref)	1 (ref)	1 (ref)
Class 3	1.45 (1.12-1.90)	1.23 (0.93-1.62)	1.84 (1.38-2.45)	1.58 (1.17-2.13)	1.90 (1.33-2.72)	1.49 (1.03-2.17)	1.63 (1.87-2.24)	1.36 (0.97-1.89)	1.78 (1.19-2.68)	1.56 (1.03-2.37)
Class 2	1.22 (0.95-1.56)	1.08 (0.83-1.41)	1.34 (1.02-1.75)	1.96 (0.90-1.60)	1.53 (1.09-2.15)	1.27 (0.88-1.81)	1.38 (1.02-1.86)	1.18 (0.86-1.62)	1.55 (1.06-2.28)	1.48 (0.99-2.21)
Class 1	1.51 (1.12-2.04)	1.10 (0.80-1.52)	1.82 (1.32-2.51)	1.34 (0.95-1.89)	2.03 (1.37-3.00)	1.33 (0.87-2-02)	1.59 (1.11-2.28)	1.10 (0.75-1.61)	2.26 (1.47-3.49)	1.85 (1.18-2.91)
***Gender***										
Male		1 (ref)		1 (ref)		1 (ref)		1 (ref)		1 (ref)
Female		2.83 (2.28-3.52)		2.62 (2.08-3.30)		2.38 (1.79-3.16)		2.94 (2.26-3.83)		1.49 (1.11-2.00)
Age at study entry in 2008		0.98 (0.92-1.03)		0.96 (0.90-1.02)		0.94 (0.87 -1.01)		1.00 (0.94-1.07)		0.86 (0.79-0.93)
***Psychological distress (HSCL-5)***										
Low level of psychological distress		1 (ref)		1 (ref)		1 (ref)		1 (ref)		1 (ref)
High level of psychological distress		3.05 (2.31-4.03)		2.78 (2.11-3.68)		3.79 (2.80-5.12)		2.75 (2.06-3.67)		2.82 (2.02-3.93)
***Education level in adulthood***										
Higher education		1 (ref)		1 (ref)		1 (ref)		1 (ref)		1 (ref)
No higher education		2.06 (1.67-2.54)		2.04 (1.64-2.54)		2.90 (1.75-2.99)		2.15 (1.69-2.74)		1.40 (1.05-1.86)

*Abbreviations: OR *Odds ratio, *AOR *adjusted odds ratio, *CPHR *Control and Payment of Health Reimbursements Registry, *HUNT *Nord-Trøndelag Health Study, *NorPD *Norwegian Prescription Database, *NPR *Norwegian Patient Registry

^a^ Shown are crude models, adjusted models and 95% CIs from logistic regression models. The first model estimates at least one recording in either of the three health registers with a drug or a diagnosis in the period 2008 to 2019, and model 1-6 estimate at least one recording for each participant in each individual health registry in the same periode. Model 7 and 8 estimates self-reported psychological distress measured with Conor Mental Health (Conor MHI) in adulthood (2017-2019 in HUNT4)

Adolescents from the high-risk group of healthy behaviours (class 1) and those from the low to moderate risk of unhealthy behaviours (class 3) had 1.51 and 1.45 higher odds of depression and/or anxiety (at least one recording in either of the three health registers with a drug or a diagnosis) than those from the low risk of unhealthy behaviours (class 4). However, these associations did not remain significant after adjustment for gender, age, psychological distress in adolescence and highest education in young adulthood in the regression analysis. According to the adjusted regression analysis of the supplemental models, adolescents from the group with low to moderate risk of unhealthy behaviours (class 3) had 1.58 (95%CI 1.17–2.13) and 1.49 (95%CI 1.03–2.17) greater likelihood of being registered with depression and/or anxiety in primary health care (CPHR [model 2]) and in specialist health care (NPR [model 4]) than those from the group with low risk of unhealthy behaviours (class 4). In addition, adolescents with low to moderate risk of unhealthy behaviours (class 3) and those with high risk of unhealthy behaviours (class 1) had 1.56 (95%CI 1.03–2.37) and 1.85 (95% CI 1.18–2.91) higher odds of self-reported psychological distress (CONOR-MHI [model 5]) than those from the group with low risk of unhealthy behaviours (class 4).

Table 4 shows the influence of gender and psychological distress on the relationship between health-related behaviours and subsequent depression or/and anxiety in primary health care (CPHR)

**Table 4:** Adjusted analysis of the moderation of gender and patterns of health-related behaviours and of psychological distress and patterns of health-related behaviours on the odds of subsequent use of depression or/and anxiety in primary health care (CPHR)

Interaction variables	CPHR	*p* value
	OR (95% CI)	
Female*classes		
Class 4	1 (ref)	
Class 3	1.65 (1.50–2.36)	0.007
Class 2	1.20 (0.85–1.71)	0.301
Class 1	1.37 (0.90–2.07)	0.144
Male*classes		
Class 4	1 (ref)	
Class 3	1.40 (0.82–2.40)	0.215
Class 2	1.13 (0.69–1.88)	0.624
Class 1	1.20 (0.65–2.22)	0.569
Psychological distress (HSCL-5 score ≤ 2.0)*classes		
Class 4	1 (ref)	
Class 3	1.44 (1.04–1.98)	0.026
Class 2	1.07 (0.79–1.46)	0.668
Class 1	1.40 (0.97–2.03)	0.074
Psychological distress (HSCL-5 score > 2.0)*classes		
Class 4	1 (ref)	
Class 3	2.88 (1.14–7.26)	0.025
Class 2	2.15 (0.90–5.11)	0.084
Class 1	1.29 (0.49–3.38)	0.607

*Abbreviations: OR *Odds ratio, *HSCL-5 *Hopkins Symptoms Checklist-5, *CPHR *Control and Payment of Health Reimbursements Registry. The outcome measure was defined as follows; at least one recording in CPHR with a diagnosis described in the period 2008–2019

Adjusted for age, psychological distress and higher education in adulthood, Adjusted for age, gender and higher education in adulthood

The results of the moderation analysis after adjustment for confounders are presented in Table 4. In general, gender and psychological distress demonstrated a moderated effect on the relationship between unhealthy behaviours (class 3) and depression or/and anxiety in primary health care. Among female adolescents in the low to moderate risk of unhealthy behaviours (class 3), reported 1.65 greater likelihood of depression and/or anxiety in primary health care than those with healthier behaviours (class 4). Considering male in the same class (class 3), the results became non-significant.

Among adolescents with psychological distress in the low to moderate risk of unhealthy behaviours (class 3), reported 2.88 greater likelihood of depression and/or anxiety in primary health care than those with healthier behaviours (class 4). Considering adolescents with lower level of psychological distress in the same class (class 3), reported 1.44 greater likelihood of depression and/or anxiety in primary health care than those with healthier behaviours (class 4).

## Discussion

To the best of our knowledge, this is the first study to longitudinally examine the patterns of health-related behaviours in adolescence and the influence of such patterns on subsequent diagnoses and/or drug treatment for anxiety and/or depression in early adulthood. In the current study, four classes of adolescents were identified, suggesting different patterns of health-related behaviours characterised by variations in physical activity level, consumption of wholegrain bread, fish, fruit, vegetables, sugar-sweetened beverages, and insomnia.

Recently, behavioural and medical sciences have been focusing on the classification of people who are likely to have specific behaviours in order to identify those individuals with different diseases risk profiles. This is in line with recent studies suggesting that lifestyle behaviours should be analysed from a multifactorial perspective by considering the different types of lifestyle behaviours together [34, 35, 64]. LCA is considered a robust method to assess different patterns of behaviours since the probability to classify the different patterns are based on specific probability method, which is less arbitrary and tend to reflect the reality more objectively. The identified patterns of health-related behaviours including low level of physical activity, low consumption of wholegrain bread, fish, fruit, vegetables and high consumption of sugar-sweetened beverages and insomnia are best interpreted in conjunction with one another. The present study has shown different patterns of health-related behaviours, ranging from the overall low-risk behaviours (class 4) to groups with moderate to high risk of unhealthy behaviours (classes 3, 2 and 1). As reported in our findings, 15.2% (class 1) of the adolescents represents the highest probability of unhealthy behaviour and 24.6% (class 4) represents those with healthiest behaviours. The biggest group of adolescents was those in class 3, 36%. Different groups of adolescents with unhealthy behaviours varied according to different probabilities of behavioural patterns (i.e., fruit and vegetables). Results are somewhat consistent with previous research that have identified four classes of multiple risk behaviours among students [39, 40].

Although crude analyses from the present study indicate an association between unhealthy behaviours in adolescence and increased odds of depression and/or anxiety in young adulthood, this association was not significant after adjustment for age, gender, and psychological distress in adolescence and educational level in young adulthood. This was observed for the main outcome, at least one recoding of the three registers, and for diagnoses in primary and specialist health care in two groups (classes 1 and 2), for prescribed drug treatment in all groups (classes 1, 2 and 3) and for self-reported psychological distress in one group (class 2). Results reported in a previous overview, however, has suggested that a healthy diet, enhanced physical activity and increased sleep time are associated with a lower risk for certain mental disorders [16]. Furthermore, a recent meta-review concluded that a healthy diet, exercise, smoking and sleep hygiene may play an important role in the prevention and treatment of mental illness [17]. Similar to our findings, a previous cross-sectional study showed that unhealthy behaviours, such as being physically inactive, having certain unhealthy eating habits and smoking tobacco and consuming alcohol, tended to co-occur among French adults in the general population, but contrary to our results, they also confirmed an association between prevalence of depression and higher risk of these unhealthy behaviours [65].

Although, results from our study did not show an association between unhealthy behaviours in adolescence and increased risk of mental illness in young adulthood, adolescents with unhealthy behaviours, including those from the low to moderate risk of unhealthy behaviours as well as those from the high-risk group of unhealthy behaviours, were more likely to self-report psychological distress in young adulthood after adjustment for psychological distress in adolescence, gender, age and education level in young adulthood. This is in line with results from a previous longitudinal study which also suggested that low levels of physical activity, high consumption of sugar-sweetened beverages, low consumption of whole grain bread and insomnia in adolescence were associated with self-reported psychological distress in young adulthood [48].

How health-related behaviours and mental health problems is operationalised and measured may have an impact on its relationship when different sample groups are compared. Both health-related behaviours and mental health problems are complex phenomena and need to be operationalised as such in research exploring the relationship between the two.

In the current study, psychological distress in adolescence was identified as the strongest predictor for subsequent depression and/or anxiety. This finding is supported by a systematic review suggesting that adolescent depression increases the risk for subsequent depression later in life and for anxiety disorders in adulthood [66]. Furthermore, the first onset of mental health disorders appears to start in childhood or adolescence and may persist throughout life [8]. These results highlight the importance of examining factors that may lower risk of developing psychological distress in adolescence and subsequent risk of depression and anxiety diagnosis later in life. Being female and without higher education was also identified as predictors for subsequent depression and/or anxiety in the current study. Gender differences related to prevalence of psychological distress has also been reported in a previous study, i.e., girls reporting significantly higher scores for psychological distress, anxiety and depression than boys [67]. Previous research has also suggested that gender differences may be explained by the fact that males may have more problems recognising their mental health problems and tend to hide their mental health problems by acting out their difficulties in more externalising problems or disorders [68]. The results from previous studies have shown that low socioeconomic status is associated with both mental health problems [69, 70] and lifestyle behaviours [71]. Thus, a reason why we did not identify a significant association between unhealthy behaviours in adolescence and mental health problems in young adulthood may be due to an already established association between lifestyle habits and mental health problems in adolescence and/or that gender differences may mask an actual association.

We did observe interaction effects in one of the models. Our findings indicate that the association between low to moderate risk of unhealthy behaviours and depression or/and anxiety in primary health care differed between males and females, and between adolescents reporting higher and lower levels of psychological distress. That is to say, the strengths of the association between low to moderate risk of unhealthy behaviours in adolescence and subsequent depression or/and anxiety in primary health care was affected by gender and psychological distress.

Several possible mechanisms may explain why other studies have identified an association between patterns of unhealthy behaviours and mental disorders. Previous research has suggested that being less interested in health and less receptive to health education messages could be one explanation for the clustering of unhealthy behaviours among those with depression [65]. Possible mechanisms explaining the observed effects of physical activity on depression and anxiety are most likely complex and might be manifested at psychological (e.g., by feelings of mastery, self-efficacy) and neurophysiological (e.g., by increasing synthesis and release of neurotransmitters, along with the neurotrophic factors associated with neurogenesis, angiogenesis and neuroplasticity) levels [72].

The mechanisms by which a proinflammatory diet and beverages could increase the risk of depression and anxiety may be through proinflammatory nutrients activating the innate immune system, which can lead to low-grade inflammation and mental health disorders [73]. Adopting an anti-inflammatory diet and diets that include omega-3-polyunsaturated fatty acids and dietary fibre might be linked to a reduced risk of developing depression and/or anxiety [74]. A recent review indicates that high dietary fibre intake may reduce inflammation by modifying both the permeability of the gut and pH levels, and a reduction in inflammatory compounds may alter neurotransmitter concentration to reduce depression [75]. Additionally, the high consumption of soft drinks has been shown to be an emotion-focused coping mechanism for mental health problems [76]. A possible biological mechanism for the association between the consumption of sugar-sweetened beverages and subsequent depression and anxiety might be related to the chronic systemic inflammation induced by sugar [73, 77].

Furthermore, sleep problems frequently co-occur with depression and anxiety [33], and results from a prospective study examining the association between sleep deprivation and depression show a reciprocal effect for major depression and sleep deprivation among adolescence [78]. Studies examining the mechanisms linking sleep and psychological distress are scarce. However, a review indicates that psychological, social and biological mechanisms underlie sleep problems and anxiety and depression in adolescence [79].

Stronger evidence on the relationships of physical activity, diet and sleep with the incidence of depression and/or anxiety is essential to improve the ability to identify those at risk of developing a mental disorder and to better inform health promotion initiatives.

As several different risky lifestyle behaviors may contribute to increase mental health problems, it is important to identify patterns of behaviors associated with mental health status, and use this information when developing health promoting programs among adolescents and young adults, i.e., in schools and health services.

### Strengths and limitations

A strength of the current study was the longitudinal design using a large and representative sample of Norwegian adolescents. The follow-up period made it possible to prospectively examine the association between patterns of unhealthy-related behaviours in adolescence and the influence of such patterns on subsequent diagnoses and drug treatment for anxiety and depression in early adulthood. Furthermore, the analyses were adjusted for well-known confounders. However, we cannot exclude possible residual confounding factors attributable to unmeasured or unknown factors, for example dietary choices (e.g., vegan, vegetarian, pescatarian, etc.) and shift workers. Measures of health-related behaviours were based solely on self-reports, which can be prone to recall bias. For example, participants tend to over-report physical activity in self-report measures when compared with objectively measured activity [80]. LCA is a powerful statistical procedure but has some limitations. The classes are based on the probabilities of being in classes given the pattern of scores they have on indicator variables [81], and because of the complexity of the classes, the name of the class does not accurately reflect the class membership and may causes ‘naming facility’ [82]. For the current study, the participants must have attended both Young-HUNTH3 and HUNT4 to be included, so there is the potential for selection bias. Previous analyses have shown that those not participating in HUNT studies have lower education and worse health [83]. A comparison of educational level between the total HUNT study population with the subpopulations included in our study reveals a further selection of individuals with higher educational levels (50% vs. 39%, respectively) [84]. In addition, information on unhealthy behaviours was not updated throughout the follow-up period. The CPHR, NPR and NorPD are complete resources for identifying contact with—and treatment in—primary health care, specialist health care and assessing prescription drug use in large populations and with the potential for long-term follow-up [85, 86]. However, it was not possible to confirm whether the dispensed drugs registered in our study reflect the actual drug use, and there was no information about the drugs used among the participants in the hospital. In addition, there was no information about possible changes in the prescription of psychotropic drugs to participants during the follow-up period.

## Conclusions

Our findings suggest that health-related behaviours are clustered among Norwegian adolescents; a small proportion of adolescents were considered with healthy behaviours. The patterns of unhealthy behaviours during adolescence only partly increased the risk of anxiety and depression in adulthood. Both health-related behaviours and mental health problems are complex phenomena and need to be operationalised as such in research exploring the relationship between the two. Additional research is fundamentally important to identify health-related behaviours that may influence mental health later in life.

## Data Availability

The HUNT Research Centre has permission from the Norwegian Data Inspectorate to store and handle these data. To protect the participants’ privacy, the HUNT Research Centre aimed to limit storage of data outside the HUNT databank and cannot deposit data in open repositories. The HUNT databank has precise information on all data exported to different projects and can reproduce these on request. There are no restrictions regarding data export given approval of applications to HUNT Research Centre. For more information, see http://www.ntnu.edu/hunt/data.

## Abbreviations

HSCL-5: Hopkins Symptoms Checklist-5
CONOR-MHI: Conor mental health index
OR: Odds ratio
HUNT: Trøndelag Health Study
CI: Confidence interval
SES: Socioeconomic status
CPHR: Control and Payment of Health Reimbursements Registry
NorPD: the Norwegian Prescription Database
NPR: the Norwegian Patient Registry
LCA: Latent class analysis
